# Molecular screening of selected RNA viruses in neotropical wild felids of Brazil

**DOI:** 10.1371/journal.pone.0359169

**Published:** 2026-10-16

**Authors:** Michelle Colpani, Aricia Duarte-Benvenuto, Carlos Sacristán, Marina Galvão Bueno, Marcia Terezinha Baroni de Moraes, Roberta Zamana-Ramblas, Bárbara Sophia Codeas, Pedro Enrique Navas-Suarez, María Alejandra Arias-Lugo, Ronaldo Gonçalves Morato, Rose Lilian Gasparini-Morato, Rodrigo Hidalgo Friciello Teixeira, Sheila Canevese Rahal, José Luiz Catão-Dias

**Affiliations:** 1 Faculdade de Medicina Veterinária e Zootecnia, Universidade Estadual Paulista, Júlio de Mesquita Filho, Botucatu, São Paulo, Brazil; 2 Faculdade de Medicina Veterinária e Zootecnia, Universidade de São Paulo, São Paulo, Brazil; 3 Grupo de Epidemiología y Sanidad Ambiental, Centro de Investigación en Sanidad Animal, INIA-CSIC, Madrid, Spain; 4 Laboratório de Virologia Comparada e Ambiental, Instituto Oswaldo Cruz, Fundação Oswaldo Cruz, Rio de Janeiro, Brazil; 5 Instituto Baleia Jubarte, Bahia, Brazil; 6 Panthera, New York, New York, United States of America; 7 Centro Nacional de Pesquisa e Conservação de Mamíferos Carnívoros, Atibaia, São Paulo, Brazil; 8 Zoológico Municipal Quinzinho de Barros, Sorocaba, São Paulo, Brazil; 9 Faculdade de Medicina Veterinária, Universidade de Sorocaba, Sorocaba, São Paulo, Brazil; Universidad Cooperativa de Colombia, COLOMBIA

## Abstract

Astrovirus, cardiovirus and rotavirus are RNA viruses widely distributed among humans, domestic and wild mammals, including felids, occasionally associated with fatal outbreaks and highly resistant to environmental conditions. Despite this, there are no reports of these viruses in New World felids. The aim of this study was to investigate the presence of astrovirus, rotavirus and cardiovirus in non-domestic felids under human care or in the wild, across multiple Brazilian biomes (i.e., Amazon Forest, Atlantic Forest, Caatinga, and Cerrado). Biological samples (e.g., blood, oral and rectal swabs, and frozen tissues) were submitted to reverse transcriptase polymerase chain reaction (RT-PCR) for astrovirus detection and real-time RT-PCR for rotavirus and cardiovirus, followed by sequencing. Fifty wild felids were analyzed, comprising 40 free-ranging and 10 captive animals: 19 ocelots (*Leopardus pardalis*), 12 pumas (*Puma concolor*), seven jaguarundis (*Herpailurus yagouaroundi*), six southern tiger cats (*Leopardus guttulus*), three margays (*Leopardus wiedii*) and three jaguars (*Panthera onca*). An astrovirus was detected in rectal swab of a healthy captive southern tiger cat, while a genotype G3P[3] *Rotavirus alphagastroenteritidis* (Rotavirus A) was detected in intestine of a roadkill puma. All felid samples were negative for cardiovirus. Morphological analyses of available formalin-fixed tissues of the rotavirus-positive puma were inconclusive due to autolysis. To the authors’ knowledge, this is the first molecular detection of astrovirus and rotavirus in Neotropical wild felid species.

## Introduction

The emergence of novel infectious disease in both human and wild animals, driven by anthropogenic environmental changes, poses significant threats to wildlife by increasing the risk of extinction in some species [1]. There is particular concern about members of the family Felidae, since over 70% of their species are facing population decline [2]. Wild felids are currently affected by several anthropogenic negative effects worldwide, including habitat fragmentation and introduction of invasive species [1]. The increasing overlap between humans, domestic, and wild animals, enhances opportunities of interspecific pathogen transmission [3]. The same scenario occurs in Brazil, where wild felids face habitat fragmentation, environmental degradation, and introduction of invasive exotic species, such as the wild boar (*Sus scrofa*) and domestic rodents (i.e., black rat [*Rattus rattus*], brown rat [*R. norvegicus*], and mouse [*Mus musculus*]) [4–6]. Wild boars are now an important component of the diet of jaguars (*Panthera onca*) and pumas (*Puma concolor*) in Brazil, while rodents are usually the most consumed prey of neotropical smaller wild felid species [2,4,7]. Both suids and rodents are reservoirs of numerous viral pathogens, including astroviruses (AstV), rotaviruses (RV) and cardioviruses [8–10].

Astroviruses (family *Astroviridae*) are single-stranded RNA viruses currently divided into two genera: *Avastrovirus*, mainly detected in avian hosts, and *Mamastrovirus* (MAstV) in mammals [11]. In humans and cats (*Felis catus*), AstV are mostly associated with gastrointestinal disease, although subclinical and neurological infections can also occur in humans and other mammalian species [12,13]. In young or immunosuppressed individuals, astroviral diarrhea can progress and become potentially fatal [12]. Similarly, rotaviruses (family *Sedoreoviridae*) are double-stranded RNA viruses, considered the main agents responsible for diarrheal outbreaks in children and frequently associated with high morbidity and mortality worldwide, with *Rotavirus alphagastroenteritidis*, commonly referred to as Rotavirus A (RVA), being the most common species detected [14,15]. Both AstV and RVA are mainly transmitted by the fecal-oral route; however, in carnivores it is important to highlight the possibility of infection via ingestion of contaminated prey [7]. Finally, cardioviruses (genus *Cardiovirus*, family *Picornaviridae*), also known as encephalomyocarditis virus (EMCV), are single-stranded RNA viruses able to induce fast and sudden death by myocarditis [2,5,7,16–18]. Their natural hosts are rodents, which are the main prey consumed by Brazilian species of wild felids [2,4,7,16,18].

Astrovirus, rotavirus and caridovirus present high recombination rates and wide host range and clinical manifestation [11,19]. Furthermore, they are non-enveloped, thus highly resistant to environmental conditions, present zoonotic potential, and have been molecularly and serologically detected in Brazil previously in humans and domestic pigs (*Sus scrofa domesticus*), mostly [12,14,20,21]. Although none of these viruses have been detected in neotropical wild felids species, they have been molecularly reported in exotic species from the Old World [19,22,23], raising concerns about their occurrence in New World felid species. Considering the relevance of wild felids for conservation for the ecosystem maintenance [24], and the lack of knowledge regarding these infectious agents in this group, the present study aims to molecularly investigate the occurrence of AstV, RVA and EMCV in wild Brazilian felids species.

## Materials and methods

### Permits

This study was approved by the Animal Ethics Committee of the School of Veterinary Medicine and Animals Science of the University of the State of São Paulo “Júlio de Mesquita Filho” (FMVZ – UNESP Botucatu) under protocol 0239/2022. Authorization for collection and transportation of biological material from non-domestic felids was granted by the Biodiversity Authorization and Information System (SISBio), identified by numbers 83450 and 76428. Finally, this study was also registered on the National System for the Management of Genetic Heritage and Associated Traditional Knowledge (SisGen) under registration code A5A3124.

### Sampling

Retrospective and prospective frozen samples collected between 2016 and 2024 from 50 wild felids of six different species were analyzed, comprising ocelot (*Leopardus pardalis*, n = 19), puma (n = 12), jaguarundi (*Herpailurus yagouaroundi*, n = 7), southern tiger cat (*Leopardus guttulus*, n = 6), margay cat (*Leopardus wiedii*, n = 3) and jaguar (n = 3). Samples were obtained from both free-ranging and captive animals including live and deceased individuals, as shown in S1 Table.

#### Free ranging animals.

Prospective samples of live free-ranging animals (n = 5) were obtained from animals inhabiting the Amazon Forest, Atlantic Forest, Caatinga, and Cerrado biomes of Brazil. In order to sample these animals, they were chemically restrained, according to the anesthetic protocols of the responsible institute performing sampling. Briefly, jaguars and pumas were restrained with 5 mg/kg of tiletamine hydrochloride and zolazepam hydrochloride (Zoletil 50, Virbac, SP, Brazil) applied intramuscularly via darts. The same application method was used for smaller felid species, chemically restrained with 12 mg/kg of 10.0% ketamine hydrochloride 0.5% (Cristália, SP, Brazil). Blood samples were stored in tubes with ethylenediaminetetraacetic (EDTA). Oral, nasal, rectal and vaginal swab samples were collected and immediately immersed in 2 mL RNase free cryotubes containing 300 µL of 10 mmol/L phosphate buffered saline. After collection, samples were frozen at −20ºC until they were transported to the Laboratory of Wildlife Comparative Pathology (LAPCOM) at School of Veterinary Medicine and Animal Science (FMVZ) of the University of São Paulo (USP), where they were stored at −80ºC. Additionally, 35 roadkill felids were analyzed, most of which were found within the state of São Paulo, in areas of Atlantic Forest, Cerrado, and transition zones, mainly on the highways BR116, SP99, SP127, and SP270. Some specimens have been included in previous studies conducted at LAPCOM [25,26]. All carcasses underwent a standardized necropsy, including collection of samples for histopathology (10.0% neutral buffered formalin) and molecular analysis (stored at −80ºC). Available retrospective frozen tissues included brain, cerebellum, lung, heart, spleen, liver, mesenteric and submandibular lymph nodes, kidneys, tonsils, skeletal muscles, and feces. Multiple tissue types per animal were analyzed whenever available, as indicated in S1 Table.

#### Animals under human care.

All animals kept under human care analyzed in this study (n = 10) came from a zoo located in an area of Atlantic Forest within the São Paulo state. The animals were sedated for routine health check-ups and samples were collected and stored as previously described. Jaguars and pumas were restrained with 10 mg/kg of 10.0% ketamine hydrochloride (Cetamin, Syntec, SP, Brazil) associated with 0.5 mg/kg of midazolam hydrochloride 0.5% (Cristália). The anesthetic protocol applied to smaller felids was the same as described above.

### Molecular analysis

For initial virus screening, target tissues were defined according to the tropism of the selected viruses and the availability of biological samples from each case, as shown in S1 Table. Total RNA was extracted from EDTA blood samples using QIamp RNA Blood Mini Kit (Qiagen, Valencia, CA, USA), and from tissues, fecal and swab samples using the RNeasy Mini Kit (Qiagen), following manufacturer’s instructions. RNase free water was used as no-template control on all extractions, which were later quantified using BioTek EPOCH equipment (Agilent, Santa Clara, CA, USA) before molecular analysis.

For AstV detection, a nested reverse transcription polymerase chain reaction (nested RT-PCR) was performed targeting fragments of 430 and 150 base pairs (bp) of the gene ORF1b. The first round of reaction was made with OneStep Enzyme (Qiagen), using primers Astr4380F and Astr4811R from a previous published protocol [19]. Four µL of each extracted RNA sample were added to a mix containing 1 µL of OneStep enzyme mix, 5 µL of Qiagen OneStep 5X, and 0.5 µL of each primer at 20 µM concentration. RNase free water was added until reaching a total volume reaction of 25 µL. This mixture was amplified in C1000 Touch Thermal Cycler (BioRad, CA, USA) with the following conditions: initial denaturation at 50ºC for 30’, reverse transcriptase reaction at 95ºC for 15’, 36 cycles of denaturation at 94ºC for 30”; annealing at 47ºC for 30”; extension at 72ºC for 30,” and a final extension at 72ºC for 7’. The second round of PCR was made by applying 4 µL of the first RT-PCR amplified product to a mix containing 0.25 µL of polymerase enzyme Platinum Taq (Invitrogen, Thermo Fisher Scientific, MA, USA) and 0.5 µL of a set of 20 µM internal primers (i.e., Astr4574F and Astr4722R) used on previously established protocol [19]. Other mixture reagents included 2.5 µL of 10X Buffer, 0.5 µL of dNTPs, 1.25 µL of MgCl_2_ and 15.5. µL of RNase free water, totalizing 25 µL of volume reaction. This mixture was amplified with the following conditions: initial denaturation at 94ºC for 5’, followed by 36 cycles of denaturation at 94ºC for 30”; annealing at 47ºC for 30”; extension at 72ºC for 30,” and a final extension at 72ºC for 7’. Samples of Human Astrovirus 1–8 extracted from human stool and previously sequenced were used as positive control. Amplified products were resolved on 1.5% agarose stained (SYBRSafe DNA Gel Stain, Thermo Fisher Scientific) gels, separated by electrophoresis and visualized in a GelDocGo Imagin System (BioRad) plate reader. Amplicons of the expected size (150 bp) were selected to be purified and sequenced. Each sample was analyzed twice under identical conditions, yielding the same results.

RVA was tested using primers, probes, and cycling conditions from a real-time PCR protocol previously published that amplifies an 80 bp fragment of gene NSP3 [27,28]. The reaction of 25 µL was made with a SuperScript^TM^ III Platinum^TM^ One-Step qRT-PCR (Thermo Fisher Scientific), using 0.5 µL of the 200 nM probe, 1 µL of each 400 nM primer and 5 µL of each sample, all amplified in QuantStudio 3 (Thermo Fisher Scientific) thermocycler. Samples with Cq values under 35.0 were considered suspected. RVA previously sequenced from human stool was used as positive control. Suspected samples were then submitted for further genotyping of VP7 and VP4 genes followed by genetic sequencing. cDNA synthesis was made separately, using a random primer hexamer according to Moraes et al. [29], with some modifications. Four µL of each cDNA were subsequently used for single-round conventional PCR protocols targeting partial fragments of 900 bp and 663 bp for genes VP7 and VP4, respectively [30]. Both reactions were performed using iTaq^TM^ DNA Polymerase (BioRad) and 0.5 µL of each primer at 20 µM, with a final volume reaction of 25 µL. Primers for both VP7 and VP4 reactions were selected according to the World’s Health Organization guidelines for RVA testing and genotyping [30]. For the VP4 reaction, the cycling conditions were the following: initial denaturation at 94ºC for 10’, followed by 40 cycles of denaturation at 94ºC for 30”; annealing at 49ºC for 30”; extension at 72ºC for 1’30,” and a final extension at 72ºC for 7’. As for VP7, initial denaturation was at 94ºC for 5’, followed by 40 cycles of denaturation at 94ºC for 30”; annealing at 50ºC for 30”; extension at 72ºC for 1’30,” and a final extension at 72ºC for 7’. Amplified products from all conventional PCRs were resolved by electrophoresis and visualized following the same conditions previously described. Each sample was tested twice upon identical conditions.

EMCV was tested using a real-time PCR that amplifies a 286 bp fragment of gene 3D, with primers described in a previously published protocol [31]. Each reaction was made with Kapa SYBR Fast Enzyme, 50X RT Kapa enzyme and ROX Reference Dye High, following concentrations suggested by the fabricant (Kapa Biosystems, Inc., Wilmington, MA, USA). Four µL of each RNA extracted sample previously diluted in RNase free water to a 1:10 proportion was added to each mix, reaching a final volume reaction of 20 µL. Amplification was performed on a StepOnePlus^TM^ Real-Time PCR System (Applied Biosystems, Foster City, CA, USA) with the following cycling conditions: reverse transcriptase reaction at 42ºC for 5’, initial denaturation at 95ºC for 3’, followed by 45 cycles of denaturation at 95ºC for 3”; annealing at 60ºC for 40”; extension at 95ºC for 15,” melting at 60ºC for 1’ and a final extension step at 95ºC for 15.” An EMCV-positive sample sequenced from heart of domestic pig was used as positive control. Samples with values of Cq lower than 35.0 and melting temperature higher than 80ºC were considered suspected.

RNase free water was used as no-template. Suspected amplified products of all reactions were purified with ExoSAP-IT PCR Product Cleanup (Affymetrix, Santa Clara, CA, USA) according to manufacturer’s instructions and later sent to Sanger sequencing in both directions.

Obtained sequences were assembled in MEGA 12.0 using ClustalW alignment and compared with those available at GenBank by BLASTn search (https://blast.ncbi.nlm.nih.gov/Blast.cgi). An AstV amino acid (aa) maximum likelihood phylogenetic tree of the RNA dependent RNA polymerase protein (49 aa) was constructed using MEGA 12.0 with a bootstrap value of 1,000 replicates. The evolutionary model Le Gascuel with Gamma distribution and Invariant sites (LG + G + I) was selected based on results of ProtTest 3.4.2 analysis. Additionally, nucleotide maximum likelihood phylogenetic trees were constructed for both RVA VP7 (778 bp) and VP4 (583 bp) genes using evolutionary model Jukes-Cantor, selected using JModel program. All bootstrap frequency values less than 70 were omitted.

### Histopathology

Histopathology was conducted on formalin-fixed samples from RT-PCR positive cases for any of the selected viral agents, if samples were available. Formalin-fixed tissues were embedded in paraffin, sectioned in 3–5 µm thickness for routine histopathological processing and stained with hematoxylin and eosin.

## Results

### Astrovirus

Total RNA extracted from 48 felids was tested for AstV. A total of 136 samples were analyzed in the first screening, prioritizing tissue samples shown in Table 1.

**Table 1:** Wild felids of Brazil and their corresponding tissue samples selected for initial astrovirus screening.

Species	N	Males		Females		Origin		Samples (n = 136)							
		A	Y	A	Y	FR	Zoo	Bl	RS	OS	Sp	ML	In	Br	Fe
*L. pardalis*	18	9	4	3	2	16	2	2	2	2	8	9	12	14	2
*P. concolor*	12	3	2	2	5	10	2	5	4	4	5	6	6	6	1
*H. yagouaroundi*	6	3	2	1	0	5	1	1	1	1	4	2	1	3	1
*L. guttulus*	6(1)	1	1	1(1)	3	5	1(1)	1	2(1)	2	3	3	4	3	2
*L. wiedii*	3	2	0	1	0	0	3	3	3	3	0	0	0	0	0
*P. onca*	3	2	0	1	0	2	1	1	2	2	0	0	0	0	0
**Total**	**48(1)**	20	9	9(1)	10	38	10(1)	13	14(1)	14	20	20	23	26	6

(): positive cases, A: Adult, Y: Young, FR: Free-ranging, Bl: Blood, RS: Rectal swab, OS: Oral swab, Sp: Spleen, ML: Mesenteric lymph node In: Intestine, Br: Brain Fe: Feces

One out of 48 (2.1%) tested wild felids was AstV positive. Specifically, one out of 136 (0.7%) samples tested positive for AstV, a rectal swab from a captive southern tiger cat. This animal was an adult female considered healthy upon clinical examination performed on the same date of sampling and maintained at a zoo, representing 10.0% (1/10) of all zoo felids tested. Upon positive result on rectal swab analysis, other samples available from the same individual (i.e., EDTA blood, oral, nasal and vaginal swabs) were tested with the same pan-AstV RT-PCR protocol, yielding negative results.

The retrieved AstV sequence was 147 bp long and presented 90.5% nucleotide identity (nt id) (Query Cover [QC]: 98.0%) with the closest available sequences in GenBank, two sequences of *Avastrovirus galli* (AAstV2) detected in chicken (*Gallus gallus*) from the United Kingdom (accession nº JN582327 and EU668998). When comparing amino acids from our AstV sequence and the available at GenBank, the closest sequence was also a AAstV2 (identity of 95.9% and a QC of 100.0%) detected in wild boar from Slovakia (accession nº AZK16184) (Fig 1). The consensus AstV sequence was submitted to GenBank under accession nº PX171445.

**Fig 1:**
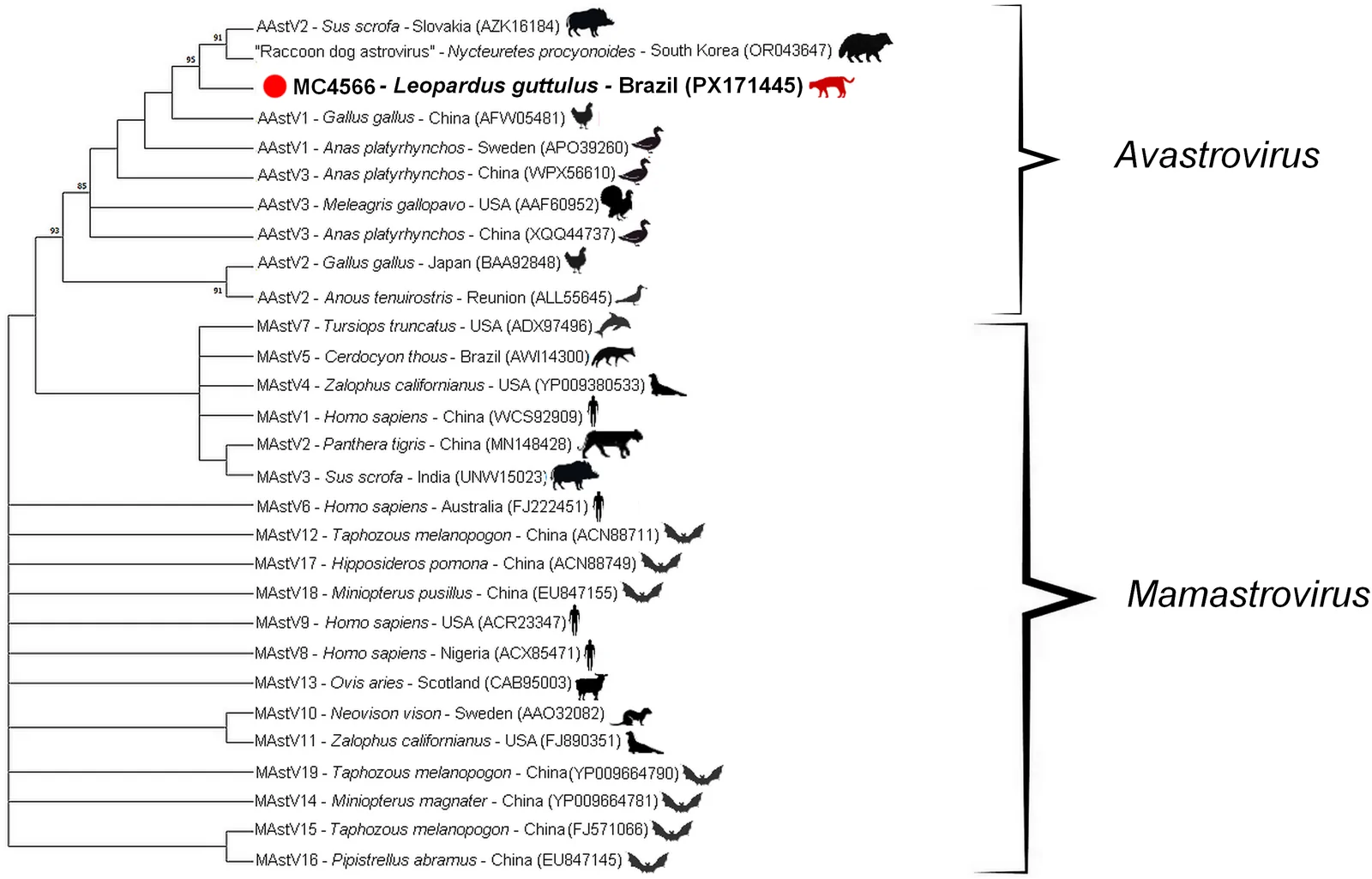
Phylogenetic analysis of partial astrovirus RNA dependent RNA polymerase protein. A phylogenetic tree based on a 49 amino acid segment was generated in MEGA 12.0 using the Maximum-Likelihood method under Le Gascuel evolutionary model with Gamma distributed rates and Invariant sites (LG + G + I) and bootstrapped with 1,000 replicates. Values under 70.0% were omitted.

### Rotavirus

A total of 47 wild felids were tested for RVA, with 58 tissues included in the initial screening (Table 2). The small intestine of one young female puma was considered positive (Cq = 28.9), accounting for 1.7% (1/58) of all samples tested. Thus, regarding all wild felids analyzed, 2.1% (1/47) were positive for RVA. This represented 9.1% of the tested pumas (1/11), 2.5% of the tested free-living wild felids (1/40) and 2.9% of the roadkill felids analyzed (1/35). Following this result, other available tissues of the same animal (i.e., trachea, heart, stomach, adrenal gland, and submandibular lymph node) were tested under identical conditions. The only sample with Cq < 35.0 was the trachea (Cq = 34.5). This puma was found in SP127 highway, in the state of São Paulo, close to Piracicaba city, a transition area of the biomes Atlantic Forest and Cerrado.

**Table 2:** Wild felids of Brazil and their corresponding tissue samples selected for initial rotavirus screening.

Species	N	Males		Females		Origin		Samples (n = 58)					
		A	Y	A	Y	FR	Zoo	Fe	RS	OS	In	ML	Li
*L. pardalis*	18	10	3	3	2	16	2	3	2	0	12	2	4
*P. concolor*	11(1)	2	2	2	5(1)	9(1)	2	1	4	0	6(1)	1	1
*H. yagouaroundi*	6	3	2	1	0	5	1	1	1	0	1	1	4
*L. guttulus*	6	1	1	1	3	5	1	2	2	0	4	0	0
*L. wiedii*	3	2	0	1	0	0	3	0	3	0	0	0	0
*P. onca*	3	2	0	1	0	2	1	0	2	1	0	0	0
**Total**	**47(1)**	20	8	9	10(1)	37(1)	10	7	14	1	23(1)	4	9

(): positive results, A: Adult, Y: Young, FR: Free-ranging, Fe: Feces, RS: Rectal swab, OS: Oral swab, In: Intestine, ML: Mesenteric lymph node, Li: Liver

The retrieved VP7 sequence was 778 bp long and presented 90.3% nt id (QC: 99.0%) with the closest sequence available at GenBank, a RVA sequence detected in laboratory Rhesus monkeys (*Macaca mulatta*) from the United States of America (USA) (HQ665466). Regarding gene VP4, a 583 bp fragment was sequenced; the closest sequence available in GenBank (nt id: 94.3%; QC: 100.0%) was an RVA P[3] sequence detected in raccoon (*Procyon lotor*) from the USA (OP947222). According to phylogenetic analysis, the retrieved VP7 sequence was classified as genotype G3 (Fig 2A), while the VP4 phylogenetic tree confirms that our sequence belonged to the P[3] genotype (Fig 2B). Both sequences were submitted to GenBank under accession numbers PX206228 (VP7) and PX136773 (VP4).

**Fig 2:**
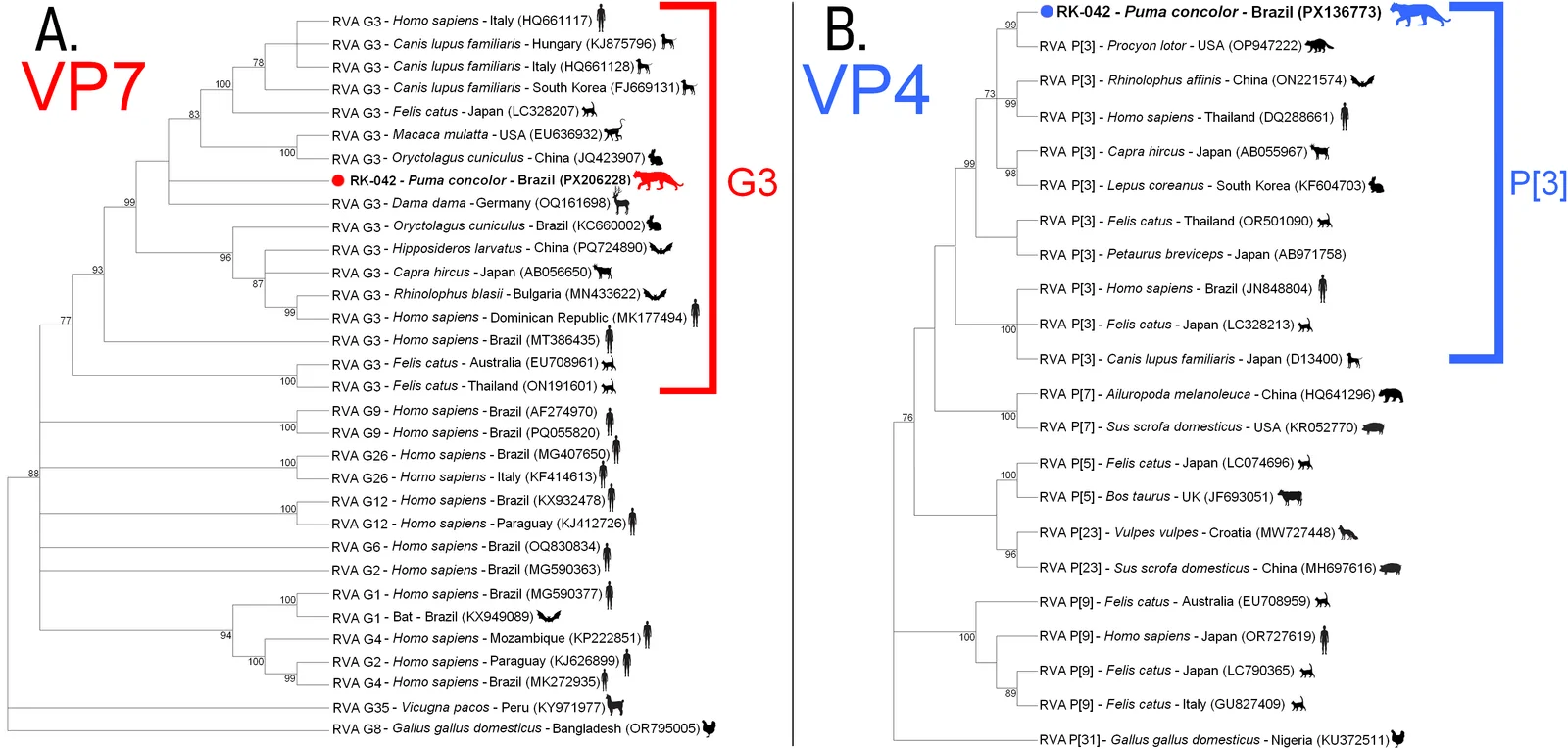
Phylogenetic analysis of the partial RVA sequence retrieved. Phylogenetic analysis was performed using MEGA 12.0 and a Maximum-Likelihood method with evolutionary model Jukes-Cantor and bootstrapped with 1,000 replicates. Values under 70.0% were omitted. 2A: Phylogenetic tree of partial (778 base pairs, bp) VP7 gene. 3B: Phylogenetic tree of partial (583 bp) VP4 gene.

Histopathology slides of small intestine were available and upon analysis revealed infection by parasites, but further diagnosis was not possible due to advanced decomposition.

### Cardiovirus

Animals and their selected target tissues for screening for cardiovirus are listed in Table 3. Overall, 134 tissues from 47 animals were tested, all cardiovirus-negative.

**Table 3:** Wild felids of Brazil and their corresponding tissues samples selected for initial cardiovirus screening.

Species	N	Males		Females		Origin		Samples (n = 134)					
		A	Y	A	Y	FR	Zoo	Bl	Br	He	Lu	Sp	Li
*L. pardalis*	19	10	4	3	2	17	2	2	13	13	12	7	12
*P. concolor*	12	3	2	2	5	10	2	5	6	5	7	4	4
*H. yagouaroundi*	7	4	2	1	0	6	1	1	3	6	4	4	5
*L. guttulus*	5	1	1	1	2	4	1	1	3	4	3	3	3
*L. wiedii*	3	2	0	1	0	0	3	3	0	0	0	0	0
*P. onca*	1	0	0	1	0	0	1	1	0	0	0	0	0
**Total**	**47**	20	9	9	9	37	10	13	25	28	26	18	24

A: Adult, Y: Young, FR: Free-ranging, Bl: Blood, Br: Brain, He: Heart, Lu: Lung, Sp: Spleen, Li: Liver

## Discussion

To the authors’ knowledge, this is the first molecular screening of astrovirus, rotavirus and cardiovirus in wild neotropical felids, including one vulnerable species, a southern tiger cat [2] kept under human care. Four different felid genera were analyzed, comprising six species that are currently undergoing population decline. There are previous reports of these viruses in Old World wild felids [19,22,23,32].

In the present study, a rectal swab of one zoo-housed southern tiger cat was positive for AstV. In domestic cats, AstV has been mainly detected in feces worldwide, with most reports identifying MAstV2 strains [33–37], which are very divergent from the retrieved sequence reported herein, since it clustered within a monophyletic branch of genus *Avastrovirus* on the phylogram. Furthermore, this sequence presented a low similarity to any MAstV2 previously detected in felids, whether domestic or wild [19,20,38].

This is not the first identification of an *Avastrovirus* in a mammalian host, which were previously reported in domestic cats, American minks (*Neovison vison*), and raccoon dogs (*Nyctereutes procyonoides*) [35,39,40]. Considering that all these species are carnivores, the detection of *Avastrovirus* was potentially related to ingestion of infected prey or contaminated food of avian origin [35,39]. In our study, apart from rectal swab, all other biological samples from the southern tiger cat were AstV-negative, suggesting ingestion of infected prey rather than systemic infection, although a localized enteric infection cannot be excluded. Similarly, *Avastrovirus* has also been detected in feces of minks presenting gastroenteritis in China [39]. Those minks were fed with chicken, likely the potential viral source [39]. In our case, the adult southern tiger cat was considered healthy upon clinical examination, not presenting any signs potentially related to AstV infection. Indeed, AstV associated diarrhea is not commonly reported in this age class, regardless of species [41]. Furthermore, AstV was not detected in any nervous tissue from wild felids.

Regarding RVA, this study reports the first detection of this virus in a wild felid species of the Americas. Specifically, small intestine sample of one young female roadkill puma was RVA RT-PCR positive. Most domestic cats RVA are classified as combinations of genotypes G3P[3] or G3P[9], with evidence of possible recombination events involving other host species [42]. Our phylogenetic analysis revealed the RVA genotype detected in this puma may correspond to the predominant detected in domestic cats (i.e., G3P[3]). This genotype is also commonly reported in several other mammal species (i.e., herbivorous, domestic rodents, and dogs), so the possibility of ingestion of an infected prey without host infection should be considered when dealing with wild carnivores, as it was previously discussed in a report with RVA-positive snow leopards (*Panthera uncia*) [21,43,44]. Reassortment and recombination events are frequent amongst RNA viruses, and they may result in novel genotypes that can be transmitted to new host species with unpredictable results [44], which could potentially explain the findings in this puma. Further studies on this virus to sequence the full FVA genotype constellation will be key to determine the evolutionary origin of the strain and to detect possible interspecies reassortment events.

Despite screening six species of wild felids and different tissue types, testing a total of 134 samples from 47 animals, and representing four different Brazilian biomes (i.e., Amazon Forest, Atlantic Forest, Caatinga, and Cerrado), cardiovirus RNA was not detected. Most cardiovirus reports in wildlife included animals kept at facilities with previous history of rodent infections [45–47]. By contrast, we tested a greater number of free-raging animals (40/50) than zoo animals (10/50). Furthermore, cardiovirus typically cause acute infections and rapid disease progression, often resulting in sudden death, and are not usually associated with long-term persistence in hosts [32,45,48]. Given that all sampled zoo wild felids were alive and healthy, the detection of cardiovirus-RNA was indeed unexpected. Additionally, this virus has not been yet reported in other species of free-raging animals apart from rodents and wild boars [8,16,49], which is in accordance with our findings. Nevertheless, there is limited knowledge on cardiovirus epidemiology in felids, evidencing the need for further molecular screening of this pathogen, particularly within areas where cardiovirus has been identified previously, including Brazil and other South American countries, such as Peru [20,50–52]. In Brazil, a cardiovirus epizootic was reported in 1960 after antibodies against EMCV were detected in wild rodents, horses, cows, birds, and mosquitoes from the Brazilian Amazon Forest [52]. Later, in 1980 there was an EMCV outbreak on a pig farm in southern Brazil, with a reemergence in 2023 on another farm in the midwest of Brazil [20], which highlights the need to further investigate this virus in different areas of Brazil. Although our research yielded negative results, we reinforce that cardiovirus should be considered as a differential diagnosis of fatal myocarditis in wildlife, especially in mammalian zoo kept species [53].

One of the study limitations was sample heterogeneity. Captive, live free-ranging, and roadkill animals were opportunistic analyzed, as well as different tissue types and samples collected across several years, which difficult epidemiological interpretations. Additionally, regarding the molecular methods adopted, neither endogenous host gene nor exogenous internal amplification control was used. Furthermore, all molecular essays were focused on an initial screening for RNA detection using pan PCR protocols that amplify conserved genes and, therefore, are not suitable for taxonomic classification at species or strain level of the viruses detected. Further studies targeting other genes are necessary for full genotyping of the AstV and RVA sequences retrieved herein. Another important limitation is the advanced state of autolysis of the RVA-positive puma. Considering it was a roadkill animal, post-mortem contamination of tissues (e.g., trachea) is a possibility, particularly as the Cq values from the trachea sample were higher (34.5) when compared to the one from intestine (Cq = 28.9). Nevertheless, histopathological examinations apart from the detections of parasites in small intestine did not offer more valuable information, also due to the advanced autolysis of the tissue. Future studies investigating pathogenicity, occurrence and full genotyping of AstV and RVA in wild felids are warranted.

## Conclusions

This is the first molecular screening for AstV, RVA and cardiovirus in wild neotropical felids. Although the pathogenicity of these viruses in felids remains to be investigated, it is important to record their molecular detection within this taxon, especially considering their role as top predators. To our best knowledge, this is the first molecular report of RVA in puma. The detected RVA strain was classified as G3P[3] based on partial VP7 and VP4 sequences. Further studies will characterize the full constellation genotype of the detected RVA. In addition, the rectal swab of a vulnerable species (the southern tiger cat) tested positive for AstV. Therefore, continuous monitoring of AstV, as well as research on their pathogenicity in wild felid species, are advised, mostly in animals under human care, since they are susceptible to stress and may develop clinical manifestations. Further sequencing is also required to characterize this astrovirus strain. Finally, to our knowledge, cardiovirus was screened for the first time in Brazilian wild felids; however, EMCV RNA was not detected in any of tested samples under the conditions and limitations (i.e., opportunistic and heterogeneous sampling) of this study.

## Supporting information

S1 Table Brazilian wild felids analyzed in this project, their available epidemiological data and description of which animal was tested for astrovirus, cardiovirus and rotavirus. AstV: Astrovirus; EMCV: Cardiovirus; RVA: Rotavirus A; A: Adult; Y: Young; C: Cub; F: Female; M: Male; M. Atl: Atlantic forest. * Indicates roadkill animals. Cities where they were found on the highways and the highways codes are noted, respectively. Predominant biomes of those cities are indicated between parentheses.
(DOCX)
