# Supplementary material for: Molecular screening of selected RNA viruses in neotropical wild felids of Brazil
Source: PLoS One. 2026 Oct 16;21(10):e0359169. doi: 10.1371/journal.pone.0359169 (PMC13674773; doi:10.1371/journal.pone.0359169)
Supplement: Supplementary file 1 [file pone.0359169.s001.docx]

**Supplementary Table 1.** Brazilian wild felids analyzed in this project, their available epidemiological data and description of which animal was tested for astrovirus, cardiovirus and rotavirus.

| ID | SPECIES | AGE | SEX | ORIGIN (BIOME) | SAMPLES | AstV | EMCV | RVA |
| --- | --- | --- | --- | --- | --- | --- | --- | --- |
| MC4890 | *P. concolor* | A | M | Salto de Pirapora (SP) (M. Atl) | Blood | X | X |  |
| MC9440 | *L. pardalis* | A | M | Zoo (M. Atl) | Blood, swab (oral, nasal, rectal) | X | X | X |
| MC3794 | *L. wiedii* | A | F | Zoo (M. Atl) | Blood, swab (oral, nasal, rectal, vaginal) | X | X | X |
| MC4769 | *L. pardalis* | A | F | Zoo (M. Atl) | Blood, swab (oral, nasal, rectal, vaginal) | X | X | X |
| MC4566 | *L. guttulus* | A | F | Zoo (M. Atl) | Blood, swab (oral, rectal, vaginal) | X | X | X |
| MC9574 | *P. concolor* | A | M | Zoo (M. Atl) | Blood, swab (oral, nasal, rectal) | X | X | X |
| MC2557 | *L. wiedii* | A | M | Zoo (M. Atl) | Blood, swab (oral, nasal, rectal) | X | X | X |
| MC3910 | *L. wiedii* | A | M | Zoo (M. Atl) | Blood | X | X | X |
| MC8105 | *P. concolor* | Y | F | Zoo (M. Atl) | Blood, swab (oral, nasal, rectal, vaginal) | X | X | X |
| CAD272.283 | *L. guttulus* | C | F | Botucatu (SP) (Cerrado/M. Atl) | Swab (oral, rectal, vaginal) | X |  | X |
| bPon543 | *P. onca* | A | M | Coronel José Dias (PI) (Caatinga) | Oral swab | X |  | X |
| VIT | *P. onca* | A | F | Zoo (M. Atl) | Blood, swab (oral, nasal, rectal, vaginal) | X | X | X |
| MC3490 | *H. yagouaroundi* | A | F | Zoo (M. Atl) | Blood, swab (oral, nasal, rectal, vaginal) | X | X | X |
| BANG | *P. concolor* | Y | F | Botucatu (SP) (Cerrado/M. Atl) | Blood, swab (oral, nasal, rectal, vaginal) | X | X | X |
| ML | *P. concolor* | Y | F | Centro Novo do Maranhão (MA) (Amazon) | Blood, swab (oral, nasal, rectal, vaginal) | X | X | X |
| bPon547 | *P. onca* | A | M | Centro Novo do Maranhão (MA) (Amazon) | Rectal swab | X |  | X |
| CS-001 | *L. pardalis* | A | M | *Caraguatatuba (SP) – SP99 (M. Atl) | Nasal swab, brain, heart, lung, liver, spleen, submandibular lymph node, kidney, intestine, feces, skeletal muscle, tonsils | X | X | X |
| CS-002 | *L. pardalis* | Y | F | *Paraibuna (SP) – SP99 (M. Atl) | Nasal swab, brain, heart, lung, liver, spleen, mesenteric lymph node, kidney, intestine, feces, skeletal muscle, tonsils | X | X | X |
| CS-009 | *P. concolor* | Y | M | *Cajati (SP) – BR116 (M. Atl) | Nasal swab, brain, heart, lung, liver, spleen, mesenteric lymph node, kidney, intestine, feces, skeletal muscle, tonsils | X | X | X |
| CS-012 | *L. guttulus* | Y | F | *Caraguatatuba (SP) – SP99 (M. Atl) | Brain, heart, lung, liver, spleen, mesenteric lymph node, kidney, intestine, feces, skeletal muscle | X | X | X |
| CS-014 | *H. yagouaroundi* | A | M | *Paraibuna (SP) – SP99 (M. Atl) | Brain, heart, lung, liver, spleen, mesenteric lymph node, kidney, intestine, feces, skeletal muscle | X | X | X |
| CS-016 | *L. guttulus* | A | M | *Caraguatatuba (SP) – SP310 (M. Atl) | Brain, heart, lung, liver, spleen, mesenteric lymph node, kidney, intestine, feces, skeletal muscle | X | X | X |
| CS-018 | *P. concolor* | A | F | *Corumbatai (SP) – SP310 (Cerrado/M. Atl) | Brain, heart, lung, liver, spleen, mesenteric lymph node, kidney, intestine, feces, skeletal muscle, tonsils | X | X | X |
| CS-019 | *L. pardalis* | A | M | *Caraguatatuba (SP) – SP99  (M. Atl) | Brain, heart, lung, liver, spleen, mesenteric lymph node, kidney, intestine, feces, skeletal muscle, tonsils | X | X | X |
| CS-025 | *L. pardalis* | A | M | *Caraguatatuba (SP) – SP310 (M. Atl) | Brain, heart, lung, liver, spleen, mesenteric lymph node, kidney, intestine, feces, skeletal muscle, tonsils | X | X | X |
| CS-026 | *L. pardalis* | Y | M | *Caraguatatuba (SP) – SP310 (M. Atl) | Brain, heart, lung, liver, spleen, mesenteric lymph node, kidney, intestine, feces, skeletal muscle, tonsils | X | X | X |
| CS-027 | *L. pardalis* | A | F | *Caraguatatuba (SP) – SP310 (M. Atl) | Brain, heart, lung, liver, spleen, mesenteric lymph node, kidney, intestine, feces, skeletal muscle, tonsils | X | X | X |
| CS-031 | *P. concolor* | A | F | *S. Miguel Arcanjo (SP) – SP331 (M. Atl) | Brain, heart, lung, liver, spleen, mesenteric lymph node, kidney, intestine, feces, skeletal muscle, tonsils | X | X | X |
| RK-032 | *L. pardalis* | Y | M | *Paraibuna (SP) – SP99 (M. Atl) | Heart, liver, spleen, mesenteric lymph node, kidney, intestine, feces | X | X | X |
| RK-034 | *P. concolor* | A | M | *Caraguatatuba (SP) – SP99 (M. Atl) | Brain, heart, lung, spleen, mesenteric lymph node, kidney, intestine | X | X | X |
| RK-039 | *L. guttulus* | Y | F | *Caraguatatuba (SP) – SP99 (M. Atl) | Heart, lung, spleen, mesenteric lymph node, kidney, intestine | X | X | X |
| RK-042 | *P. concolor* | Y | F | *Piracicaba (SP) – SP127 (Cerrado/M. Atl) | Heart, lung, intestine | X | X | X |
| RK-062 | *H. yagouaroundi* | Y | M | *Jambeiro (SP) – SP99 (M. Atl) | Brain, heart, liver, mesenteric lymph node | X | X | X |
| RK-066 | *L. pardalis* | A | M | *Caraguatatuba (SP) – SP99 (M. Atl) | Brain, lung, liver, mesenteric lymph node, kidney | X | X | X |
| RK-067 | *L. pardalis* | Y | M | *Miracatu (SP) – BR116  (M. Atl) | Brain, heart | X | X |  |
| RK-088 | *H. yagouaroundi* | A | M | *Atibaia (SP) – BR381 (M. Atl) | Heart, lung, liver, spleen, and kidney | X | X | X |
| RK-089 | *H. yagouaroundi* | Y | M | *Atibaia (SP) – BR381 (M. Atl) | Heart, lung, liver, spleen, kidney | X | X | X |
| RK-104 | *H. yagouaroundi* | A | M | *Atibaia (SP) – BR381 (M. Atl) | Heart |  | X |  |
| RK-175 | *L. pardalis* | A | F | *Caraguatatuba (SP) – SP99 (M. Atl) | Brain, heart, lung, liver, kidney, | X | X | X |
| RK-205 | *H. yagouaroundi* | A | M | *Paraibuna (SP) – SP99 (M. Atl) | Brain, heart, lung, liver, spleen, kidney | X | X | X |
| RK-206 | *P. concolor* | Y | F | *Registro (SP) – BR116  (M. Atl) | Brain, lung, liver, mesenteric lymph node, kidney | X | X | X |
| RK-263 | *L. pardalis* | Y | M | *Ipaussu (SP) – SP270 (M. Atl) | Brain, heart, lung, liver, mesenteric lymph node, kidney | X | X | X |
| RK-273 | *L. pardalis* | Y | F | *Duque de Caxias (RJ) – BR116 (M. Atl) | Brain, heart, lung, mesenteric lymph node, kidney, intestine | X | X | X |
| RK-276 | *L. pardalis* | A | M | *Cajati (SP) – BR116 (M. Atl) | Brain, lung, intestine | X | X | X |
| RK-278 | *L. pardalis* | A | M | *Duque de Caxias (RJ) – BR116 (M. Atl) | Brain, heart, lung, liver, spleen, mesenteric lymph node, kidney, intestine | X | X | X |
| RK-280 | *P. concolor* | Y | M | *Cajati (SP) – BR116 (M. Atl) | Brain, lung, spleen, intestine | X | X | X |
| RK-303 | *L. guttulus* | Y | M | *Paraibuna (SP) – SP99 (M. Atl) | Brain, heart, liver, mesenteric lymph node, kidney, intestine | X | X | X |
| RK-310 | *L. pardalis* | A | M | *Caraguatatuba (SP) – SP99 (M. Atl) | Liver |  | X | X |
| RK-400 | *L. pardalis* | A | M | *Paraibuna (SP) – SP99 (M. Atl) | Brain, heart, lung, liver, mesenteric lymph node, kidney, intestine | X | X | X |
| RK-414 | *L. pardalis* | A | M | *Paraibuna (SP) – SP99 (M. Atl) | Heart, kidney, intestine, skeletal muscle | X | X | X |

AstV: Astrovirus; EMCV: Cardiovirus; RVA: Rotavirus A; A: Adult; Y: Young; C: Cub;

F: Female; M: Male; M. Atl: Atlantic forest

* Indicates roadkill animals. Cities where they were found on the highways and the highways codes are noted, respectively. Predominant biomes of those cities are indicated between parentheses.
